# List of reviewers 2021

**DOI:** 10.1192/bjb.2022.21

**Published:** 2022-06

On behalf of the editorial board, the Editor-in-Chief would like to thank the following for their contribution as peer reviewers in the period from January 2021 – December 2021. Their anonymous work for the journal forms the foundation to its success, and is highly appreciated.

Mohammed Abousaleh

Alex Acosta-Armas

Gwen Adshead

Suhana Ahmed

Mike Akroyd

Adam Al-Diwani

Kirstie Anderson

Stephen Anderson

Humma Andleeb

Neil Armstrong

Mark Ashworth

Oyedeji Ayonrinde

Agnes Ayton

Harriet Ball

Zainab Bashir

Nick Bass

Chloe Beale

Rachel Bingham

Harm Boer

Lana Bojanić

Philippa Bolton

Mary Bosworth

Tim Bradshaw

Helen Bruce

Tom Burns

Peter Byrne

Elspeth Carruthers

Emanuele Castano

Erica Cini

Tim Clarke

Seonaid Cleare

Kristin Cleverley

Katherine Clyde

Alastair Cockburn

Lucy Colbourne

John Cookson

Ken Courtenay

John Cox

Helen Crimlisk

Adam Croom

Pim Cuijpers

Stephen Curran

David Curtis

Phil Davison

Michael Deckard

Mayura Deshpande

Jack Drescher

Lynne Drummond

Chantal Edge

George El-Nimr

Carolina Estevao

Dominic Fannon

Robert Flanagan

Akira Fukutomi

Niall Galbraith

Chris Garrett

Linda Gask

Michael Goldacre

Philip Graham

William Grant

William Grant

Neel Halder

Nicholas Hallett

Guy Harvey

Matthew Hotopf

Allan House

Ranjita Howard

Ahmed Huda

Rebekah Humphreys

Nusrat Husain

Nusrat Husain

Eduardo Iacoponi

George Ikkos

Jeremy Isaacs

Eric Jarvis

Sameer Jauhar

Afzal Javed

Louise Johns

Fiona Johnstone

Edgar Jones

Menna Jones

Nikolina Jovanovic

Anju Keetharuth

Alan Kellas

Brendan Kelly

David Kessler

Michael King

Martin Knapp

Richard Latham

John Launer

Stephen Lawrie

Tennyson Lee

Belinda Lennox

Janine Limoncelli

Joanne Lloyd

Yona Lunsky

Oscar Lyons

Fiona Mathieson

Lynsey McAlpine

Rosalind McAlpine

Lynsey McAlpine

Mary McMurran

C Mizen

Azer Mohammed

Paul Moran

Santoshkumar Mudholkar

Caz Nahman

Rajan Nathan

David Ndetei

Louise Newman

Giles Newton-Howes

David O' Regan

Aileen O'Brien

Mary O'Kane

Chris O'Loughlin

Femi Oyebode

Carmine Pariante

Caroline Parker

Carol Paton

Steve Pearce

Catherine Penny

Giulia Piazza

Alexandra Pitman

Norman Poole

Rob Poole

Annabel Price

Keith Reid

Antonia Rich

Ruth Riley

Keith Rix

Jonathan Rogers

Ivana Rosenzweig

Ross Runciman

Howard Ryland

Abdi Sanati

Karl Scheeres

Samantha Scholtz

Sharmita Sharmacharja

Rory Sheehan

David Skuse

Richard Smith

Timothy Smith

Daniel Smith

Sujata Soni

Ketan Sonigra

Pavan Srireddy

Faye Stanage

Thomas Steare

Anne Stewart

Sarah Sullivan

Derek Summerfield

Su-Gwan Tham

Alex Thomson

Alex Till

Derek Tracy

Peter Tyrer

Benjamin Underwood

David Veale

Krishnaveni Vedavanam

John Warren

Lauren Waterman

Richard Weiner

George Zaharias

Anli Zhou

